# Laser Self-Mixing Fiber Bragg Grating Sensor for Acoustic Emission Measurement

**DOI:** 10.3390/s18061956

**Published:** 2018-06-16

**Authors:** Bin Liu, Yuxi Ruan, Yanguang Yu, Jiangtao Xi, Qinghua Guo, Jun Tong, Ginu Rajan

**Affiliations:** School of Electrical, Computer and Telecommunications Engineering, University of Wollongong, Northfields Avenue, Wollongong, NSW 2522, Australia; bl987@uowmail.edu.au (B.L.); yr776@uowmail.edu.au (Y.R.); jiangtao@uow.edu.au (J.X.); qguo@uow.edu.au (Q.G.); jtong@uow.edu.au (J.T.); ginu@uow.edu.au (G.R.)

**Keywords:** laser diode, acoustic emission measurement, fiber Bragg grating, self-mixing interferometry

## Abstract

Fiber Bragg grating (FBG) is considered a good candidate for acoustic emission (AE) measurement. The sensing and measurement in traditional FBG-based AE systems are based on the variation in laser intensity induced by the Bragg wavelength shift. This paper presents a sensing system by combining self-mixing interference (SMI) in a laser diode and FBG for AE measurement, aiming to form a new compact and cost-effective sensing system. The measurement model of the overall system was derived. The performance of the presented system was investigated from both aspects of theory and experiment. The results show that the proposed system is able to measure AE events with high resolution and over a wide dynamic frequency range.

## 1. Introduction

Acoustic emissions (AEs) are the transient elastic waves within a material, caused by the rapid release of localized stress energy. AE testing is a well-known technique in detecting stress/strain waves generated by structural defects, allowing continuous structural monitoring during the service life of an infrastructure [1,2,3]. Traditionally, piezoelectric crystal transducers are used to detect AE events. In recent decades, optical fiber based AE detection is getting wide acceptance because fiber-optic sensors offer many benefits compared with their electric counterparts, e.g., immunity to electromagnetic interference, low cost, and capability of directly attaching or embedding in the host structure without modifying the host’s properties and functions while maintaining the structural integrity [4]. Amongst the varieties of fiber-optic sensors, fiber Bragg gratings (FBGs) are considered as the most popular technology for implementing health-monitoring systems because in addition to the advantages mentioned above, FBG offers some other advantages, e.g., ease of multiplexing, and simultaneous measurement of several parameters such as temperature and strain [5,6]. Traditionally, an FBG interrogation system is needed to detect the reflected wavelength shift induced by external parameters on the FBG such as strain and temperature. There are two main challenges when using FBG for AE testing. Firstly, the typical frequency of an AE event is from a few KHz to several MHz which requires a wide dynamic measurement range. Secondly, the related strain level caused by an AE event is normally in the micro-strain scale [7]. However, conventional FBG interrogation systems have relatively small measurement frequency bandwidth and the strain sensitivity is not sufficient to measure the AE induced events. To measure the AE induced strains in FBG, high sensitivity and bandwidth interrogation systems are required. Recently, Rajan et al. [6] demonstrated the capability of a commercial distributed fiber optic acoustic emission sensor (FAESense) interrogation system to detect the acoustic emission in ballast crack activities. The above system requires an expensive interrogator which makes the overall system sophisticated and costly.

Recently, some low cost FBG-based AE systems were reported for different applications. C. Baldwin et al. presented an FBG-based acoustic emission crack detection system by using a matched FBG as a passive optical filter for the reflected signal from the sensing FBG [8]. J.R. Lee et al. designed an FBG–AE sensor head for mechanical test with a wide temperature operation range [9]. H. Tsuda et al. measured the AE during a pressure test of carbon fiber-reinforced plastic tank by using an FBG [10]. N. Mabry et al. measured the AE Felicity ratio in a carbon composite structure by using FBG [11]. Raju et al. presented an FBG-based AE detection technique for the failure characterization of the top-hat stiffener [12]. Q. Wu designed a phase-shifted FBG balancing sensing system [13], which was used for detecting AE induced by damages in CFRP laminates [14,15]. The sensing and measurement in these systems are based on the variation in laser intensity induced by the Bragg wavelength shift. In order to achieve a good linearity and wide measurement range, an operation point for these systems, e.g., the 3-dB position of the absorption filters transmittance, is needed to be set. Additionally, for these systems, an external photodetector is required.

By combing self-mixing interference (SMI) and FBG technology, we aimed to build a new compact and cost-effective system meanwhile capable of achieving a wide dynamic measurement range. SMI is an emerging non-destructive sensing technology for measuring parameters such as distance, displacement, and velocity which has attracted intensive research because of its merits of compact structure, low-cost, simple implementation, and high resolution [16,17,18,19,20,21]. Self-mixing effect in a laser diode (LD) occurs when a fraction light emitted by a laser diode is reflected by an external target and re-enters the laser active cavity. In this case, both the frequency and intensity of the emitted laser can be modulated. The modulated laser intensity is usually called the SMI signal, which can be used for measuring metrological quantities of the target and the parameter of laser diode itself [22,23,24]. In this work, a 3 m long fiber with an FBG attached to its one end is used as the external cavity of an LD. FBG is the target and the dynamic strain source replicates an acoustic emission event and the self-mixing LD converts the strain within the fiber containing the FBG to an SMI signal.

Regarding the strain measurement by SMI method, D. Tosi reported a chaotic SMI system [25,26]. M. Suleiman et al. [27,28] demonstrated an SMI system with weak feedback level for measuring dynamic strains. By considering the features of AE events and based on our previous design on AE measurement [6], we describe a complete measurement model to show the relationship between an SMI signal and the equivalent dynamic deformation applied on an FBG caused by an AE signal. The model described is suitable for different feedback levels including a weak, moderate, and even a strong feedback case. Meanwhile, a varying refractive index of the FBG is considered and included in the model, which makes the measurement model more accurate. By correctly setting the LD and making its emitting spectrum match the reflective spectrum of the FBG, high quality SMI signals were obtained by the experimental system designed in this paper. We also designed the system to detect AE waves. In the experiments, a 40 KHz ultrasonic transducer and pencil lead breaking were used as the AE source. In this design, the broad band laser source and interrogation system required by existing FBG-based AE measurement system can be removed and a wide dynamic measurement range can be achieved.

## 2. The Model of FBG–SMI System

### 2.1. Schematic of an FBG–SMI System

**Figure 1:**
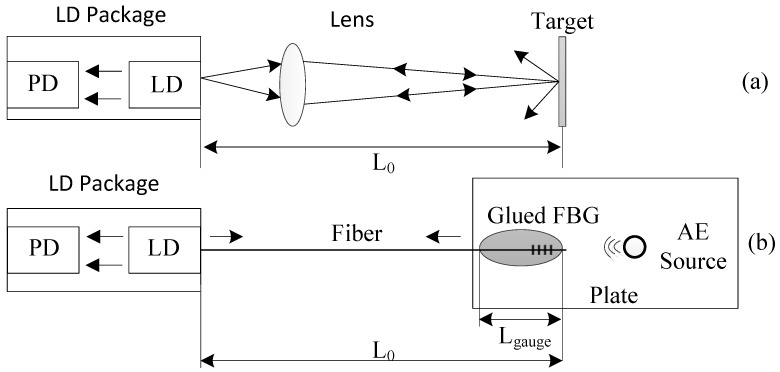
Schematic diagram of a self-mixing interference (SMI) system, (**a**) typical SMI system, (**b**) fiber Bragg grating (FBG)–SMI system.

The sensing mechanism of an SMI system is based on the self-mixing effect in a laser. Similar to Michelson interferometry, the laser intensity in an SMI system is modulated in the form of interference fringes due to mixing of the intra-cavity electromagnetic wave with an emitted electromagnetic wave re-injected into the laser cavity after interaction in the external cavity. The external strain causes the change in the light path, and thus a self-mixing interferometric signal can be generated. Figure 1 shows the schematic of a typical SMI system (Figure 1a) and an FBG–SMI system (Figure 1b). A typical SMI system consists of an LD, a photodiode (PD) attached to the LD, a lens and an external target. Instead of the free space between the laser front facet and the target in a typical SMI system, in an FBG–SMI system, a piece of fiber with length of $L_{0}$ with an FBG constitutes the external cavity. Because the most basic form of an FBG is a periodic modulation of the refractive index along a single mode fiber, the light phase in an FBG–SMI system will be different from that in the typical SMI system. The FBG is a distributed reflector, which usually acts as a narrowband reflection filter. The maximum reflectivity of the FBG occurs at a wavelength matching the Bragg condition [5]:

(1) $$\lambda_{B}=2n_{eff}\Lambda ,$$

where $\lambda_{B}$ is the peak reflected wavelength, $n_{eff}$ is the effective refractive index of the fiber, and Λ is the grating pitch [29]. FBG sensors are usually used for temperature or strain measurement. When the temperatures varies or a longitudinal stain is imposed on the FBG, both the $n_{eff}$ and Λ will be changed, resulting in the wavelength shift. By monitoring the shift of $\lambda_{B}$, the temperature change or strain can be measured. In the following derivation of the measurement model, we assume that an AE event causes a dynamic strain change and the environmental temperature is constant.

### 2.2. Measurement Model of an FBG–SMI System

The behavior of an LD with external optical feedback can be described by the well-known Lang–Kobayashi (L–K) equations [30]. The widely accepted sensing model for a typical SMI system is derived from the L–K equations by solving its steady state solution, which is described as follows [31,32]:

(2) $$\phi_{0}(t)=4\pi nL(t)/\lambda_{0}$$

(3) $$\phi_{F}(t)=\phi_{0}(t)-C\sin(\phi_{F}(t)+\arctan\alpha )$$

(4) $$g(t)=\cos(\phi_{F}(t))$$

(5) $$P(t)=P_{0}(1+m\times g(t))$$

where, $\lambda_{0}$ is laser wavelength without external optical feedback, α is the linewidth enhancement factor, $\phi_{F}(t)$ and $\phi_{0}(t)$ are the external light phases at the location of the target for the LD with and without feedback respectively, n = 1 for Figure 1a, which is the refractive index of air. *L*(*t*) is the instant external cavity length, which is expressed as $L(t)=L_{0}+\Delta L(t)$, where, $L_{0}$ is the initial external cavity length, and ΔL(t) is the varying part caused by a physical quality to be measured, e.g., displacement, velocity, or vibration. From an SMI system, P(t) can be observed and is called the SMI signal. In Equation (5), $P_{0}$ is the power emitted by the free running LD, m is the modulation index (with typical values ~10^−3^). The normalized SMI signal g(t) can be obtained from P(t) by normalizing it. Since a physical quantity to be measured is generally linked to ΔL(t), thus causing a phase change in $\phi_{0}(t)$ shown by (2). For an SMI based sensing scheme, $\phi_{0}(t)$ needs to be extracted from P(t) following the procedure: $P(t)\to g(t)\to \phi_{F}(t)\to \phi_{0}(t)$. In (3), C is the feedback coefficient, which is defined by (6):

(6) $$C=\eta (1-{r_{2}}^{2})(\frac{r_{3}}{r_{2}})\sqrt{1+\alpha^{2}}\frac{\tau }{\tau_{in}}$$

where, $r_{2}$ is the amplitude reflection coefficient of the front facet of the LD, and $r_{3}$ is the amplitude reflection coefficient of the front facet of the external target, $\tau_{in}$ is the internal roundtrip time determined by the length and refractive index of laser internal cavity, τ is the external roundtrip time of light transmitting in the external cavity. η is the coupling efficiency and accounts for possible loss on re-injection. In the case of optical fiber as the external cavity, the length of the fiber is long and it makes C larger [33]. Therefore FBG–SMI system can easily enter moderate feedback case with C > 1. For an FBG–SMI system, when a strain is applied on the FBG, $r_{3}$ may change, which may lead to the feedback coefficient C changing. Thus, it may cause fluctuation in the amplitude of the SMI signals which is similar to the effect of speckle as shown in [34]. In this case, extra signal processing like that in [34] is needed to eliminate the fluctuation. In this work, in order to reduce the effect of strain-induced FBG wavelength shift, we choose the FBG with a wider full width at half maximum (FWHM) than the emitting spectrum of the LD. Additionally, we make the LD spectrum locate at the center of the reflective spectrum of the FBG to obtain a wide flat response range. By doing so, we can keep C nearly constant during the measurement.

For the FBG–SMI system shown in Figure 1b, the laser is coupled into an optical fiber. The external cavity is the optical fiber with the FBG at one end. The gauge length is the length of the fiber with the FBG glued on the plate for sensing the AE-induced strain, denoted by $L_{gauge}$. The length of the fiber including the FBG is the initial external cavity length, denoted by $L_{0}$. Once AE occurs, a corresponding dynamic strain on the FBG (denoted by ε(t)) is expressed by $\varepsilon (t)=\Delta L(t)/L_{gauge}$, where ΔL(t) is the dynamic deformation of the gauge fiber with FBG. The deformation will make changes in both physical length and refractive index of the gauge fiber with FBG. The two factors will thus modify the optical phase $\phi_{0}(t)$ as below. Suppose the original effective refractive index of the FBG is $n_{eff0}$, the instant $n_{eff}(t)$ is expressed as $n_{eff}(t)=n_{eff0}+\Delta n_{eff}(t)$, where $\Delta n_{eff}(t)$ is the varying part caused by a dynamic strain. The instant external cavity is $L(t)=L_{0}+\Delta L(t)$.

(7) $$\begin{array}{cc} \phi_{0}(t) & =\frac{4\pi n_{eff0}(L_{0}-L_{gauge})}{\lambda_{0}}+\frac{4\pi (L_{gauge}+\Delta L(t))(n_{eff0}+\Delta n_{eff}(t))}{\lambda_{0}}\\ & =\frac{4\pi n_{eff0}L_{0}}{\lambda_{0}}+\frac{4\pi \Delta L(t)n_{eff0}}{\lambda_{0}}+\frac{4\pi L_{gauge}\Delta n_{eff}(t)}{\lambda_{0}}+\frac{4\pi \Delta L(t)\Delta n_{eff}(t)}{\lambda_{0}} \end{array}$$

where the last term $4\pi \Delta L(t)\Delta n_{eff}(t)/\lambda_{0}$ is much smaller than the other terms, thus it can be neglected. Thus the optical phase can be written as:

(8) $$\phi_{0}(t)\approx \frac{4\pi n_{eff0}L_{0}}{\lambda_{0}}+\frac{4\pi \Delta L(t)n_{eff0}}{\lambda_{0}}+\frac{4\pi L_{gauge}\Delta n_{eff}(t)}{\lambda_{0}}$$

Due to the photo-elastic effect, the refractive index of the fiber containing the FBG under a strain can be expressed as (9) assuming the temperature as constant [2,3]:

(9) $$n_{eff}[\varepsilon (t)]=n_{eff0}[1-p_{e}\varepsilon (t)]$$

(10) $$\Delta n_{eff}(t)=-p_{e}\Delta L(t)/L_{gauge}$$

where, μ is the Poisson’s ratio, $p_{11}$, $p_{12}$, are Pockel’s strain-optic tensor coefficients, which are all constants for a specific FBG, $p_{e}={n^{2}}_{eff0}[p_{12}-\mu (p_{11}+p_{12})]/2$ is known as the effective photo-elastic constant. Substituting (10) to (8), we get:

(11) $$\begin{array}{cc} \phi_{0}(t) & =\frac{4\pi n_{eff0}}{\lambda_{0}}L_{0}+\frac{4\pi n_{eff0}(1-p_{e})}{\lambda_{0}}\Delta L(t)\\ & =\phi_{00}+\Delta \phi_{0}(t) \end{array}$$

where, $\phi_{00}$ is the initial light phase for a stationary target, $\Delta \phi_{0}(t)$ is the varying part in the light phase correlated to the dynamic strain applied on the FBG. Therefore, Equations (3)–(5) and (11) are considered as the measurement model for an FBG–SMI system. The AE induced dynamic strain is applied on the FBG–SMI and causes a modulated laser intensity (called SMI signal) following this procedure: $\varepsilon (t)\to \Delta L\to \phi_{0}(t)\to \phi_{F}(t)\to g(t)\to P(t)$. We can then retrieve the strain through the observed P(t).

**Figure 2:**
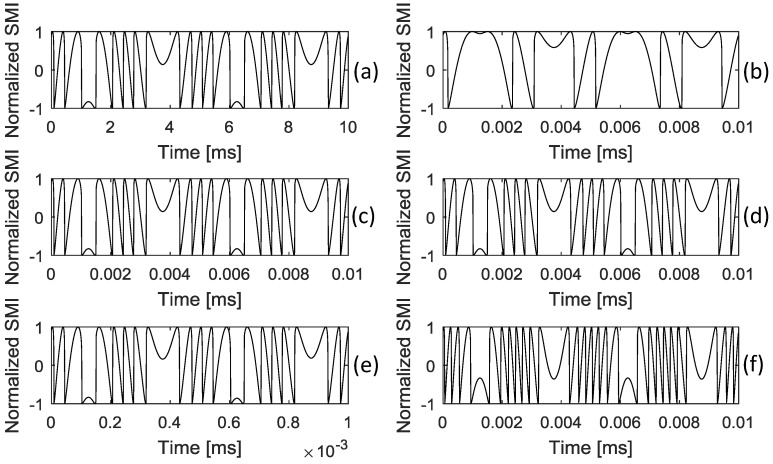
Responses of the FBG–SMI system to strains with different frequencies and amplitudes, (**a**,**c**,**e**) show the normalized SMI signals for a dynamic strain with 20.7 με magnitude respectively at frequency of 200 Hz, 200 KHz, and 2 MHz; (**b**,**d**,**f**) show the normalized SMI signals for a dynamic strain at 200 KHz but with the magnitudes as 10.3 με, 20.7 με, and 31.0 με respectively.

From (11), it can be seen that the change of the equivalent optical path length in a FBG–SMI system is $n_{eff0}(1-p_{e})\Delta L(t)$. When the equivalent light path length change is $\lambda_{0}/2$, that is $n_{eff0}(1-p_{e})\Delta L(t)=\lambda_{0}/2$, the corresponding SMI signal g(t) has a fringe change. For the FBG we used, we have the parameters as $p_{11}=0.113$, $p_{12}=0.252$, ,μ=0.16 and $n_{eff0}=1.48$ [29], so we can have each fringe change corresponding to the deformation of ΔL(t) with 0.429*λ*_0_. It can be seen from Equation (2) that ignoring the varying refractive index can result in an absolute error with 0.091*λ*_0_ in each fringe for deformation measurement (ΔL(t)) as seen in the work presented in [27,28]. Therefore, it can be argued that including the change of refractive index in the measurement model presented in this paper is more accurate than the existing model. The LD we used has its wavelength *λ*_0_ with 1550 nm. The gauge length $L_{gauge}$ is 15 cm. We can say the strain resolution is 4.4 με by the fringe counting method. The resolution can be further improved by the waveform reconstruction algorithm reported in [31,32]. Regarding the dynamic response, the maximum response speed for an SMI system depends on the photodiode (PD) and the related detection circuit. Our current physical experimental system has a bandwidth with 10 MHz. This fits well for AE measurement [1]. Simulations are made in Figure 2 to show that the proposed FBG–SMI sensor has a wide frequency response and good sensing relationship between the strain measured and the SMI signal. The left column in Figure 2, i.e., (a), (c), and (e), shows the normalized SMI signals for a dynamic strain with the same magnitude of 20.7 με but at different frequencies respectively with 200 Hz, 20 KHz, and 2 MHz. It can be seen that the FBG–SMI has the same response for this set of strain signals covering a large range of frequency. The right column in Figure 2 shows the normalized SMI signals corresponds to a set of strain signals operating at same frequency of 200 KHz but different magnitudes respectively with 10.3 με, 20.7 με, and 31.0 με. The fringe number in the SMI signal increases with the strain magnitude. There is a linear relationship between the stain magnitude and the fringe number.

## 3. Experiment

### 3.1. Verification of the Proposed Model

**Figure 3:**
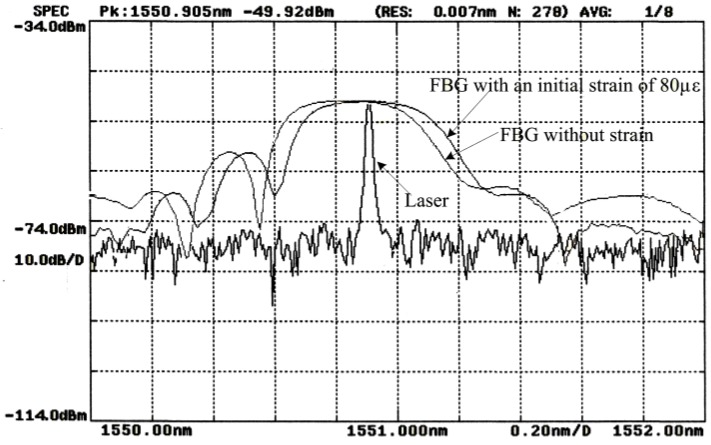
Optical spectra respectively for the laser diode (LD) and FBG with and without initial strain.

To obtain a clear SMI signal from an FBG–SMI system and have a signal with approximate constant amplitude, first, the reflective spectrum of the FBG should have a wider FWHM than the emitting spectrum of the LD. The peak of the LD spectrum should locate at the center of the reflective spectrum of the FBG. This can be achieved by carefully and accurately adjusting the injection current to the LD and testing its emitting spectrum. Meanwhile, an initial strain can be applied on the FBG to adjust its reflective spectrum so that the two spectra can achieve an optimal match, as shown in Figure 3. In our experiments, a distributed feedback (DFB) laser diode with pigtail with a wavelength of 1550 nm was chosen as the laser source. The length of the FBG used was 3 mm with an FWHM of ~0.5 nm and a reflectivity greater than 80%. The typical values of other parameters for the FBG are: $p_{11}=0.113$, $p_{12}=0.252$, μ=0.16, and $n_{eff0}=1.48$. As shown in Figure 3, a pre-strain of 80 με is applied to the FBG to ensure that the LD signal lies at the center of the FBG signal with an injection current of 25 mA applied to the LD at a temperature of 20 °C.

**Figure 4:**
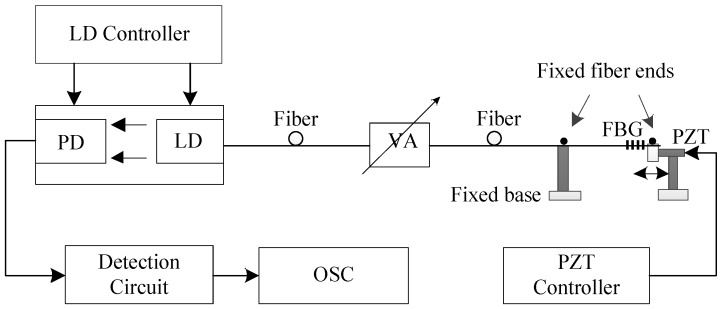
Experimental FBG–SMI system for verifying the proposed model: LD, laser diode; PD, photodiode; VA, variable attenuator; PZT, piezoelectric transducer; OSC, oscilloscope.

To verify the proposed measurement model described in Section 2, an FBG–SMI experimental system was built and is depicted in Figure 4. The LD is a DFB laser diode with pigtail (LP1550-SAD2, Thorlabs, Newton, NJ, USA) with a wavelength of 1550 nm and maximum output power of 2 mW. The LD is driven by a combined laser diode and temperature controller (ICT4001, Thorlabs, Newton, NJ, USA), operating at injection current of 25 mA (threshold current is 10 mA) and temperature of T=20±0.01 °C. An optical variable attenuator (VOA50, Thorlabs, Newton, NJ, USA) is applied to adjust the optical feedback level. The PD attached to the LD is used to convert the laser intensity to an electrical signal and then the signal is further processed by the detection circuit and then sent to the oscilloscope for display. In the experiments, we use a piezoelectric transducer (PZT) to generate the dynamic strain on the FBG. One end of the FBG is glued on a fixed base and the other end is glued on a PZT. The PZT (PAS009, Thorlabs, Newton, NJ, USA) is controlled by a PZT controller (MDT694, Thorlabs, Newton, NJ, USA) which can be used to generate dynamic longitudinal strains along the FBG.

**Figure 5:**
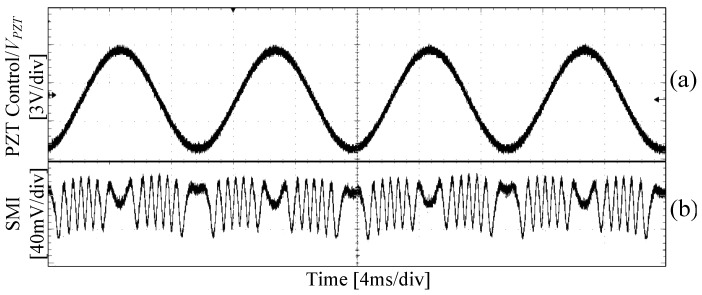
SMI signal of the FBG–SMI system when *L*_0_ = 3 m, I = 25 mA, (**a**) Control voltage applied on PZT, (**b**) SMI signal.

The PZT controller is used to adjust the control voltage signal (denoted by $V_{PZT}$) of the PZT. Each 1 V change in $V_{PZT}$ causes the PZT to have a 530 nm displacement. The maximum displacement of the PZT is 40 μm with a resolution of 40 nm. In the experiments, the initial external cavity length $L_{0}$ is set as *L*_0_ = 3 m, and the gauge length *L_gauge_* is set as *L_gauge_* = 15 cm. The control voltage signal applied on the PZT is a sinusoidal signal, i.e., $V_{PZT}=4\sin\left(200\pi t\right)$. The observed SMI signal is shown in Figure 5, (a) is the PZT control signal, and (b) is the corresponding SMI signal. From Figure 5, it can be seen that there are 6.5 fringes within a half oscillation period, corresponding to 4322 nm (0.429 × 1550 nm × 6.5, as we discussed in Section 2 by using the typical values of the FBG’s parameters, each fringe corresponds to 0.429*λ*_0_) which is equivalent to a strain of 28.8 με.

**Figure 6:**
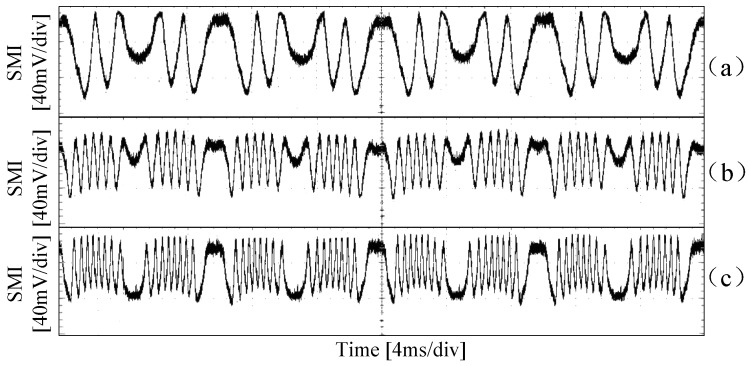
SMI signals for VPZT with frequency of 100 Hz but different amplitudes. (**a**) $V_{PZT}=3.6\text{ V}$, (**b**) $V_{PZT}=8.0\text{ V}$, (**c**) $V_{PZT}=11.0\text{ V}$.

We also changed the amplitude of the control voltage signals on the PZT. Figure 6 shows the SMI signals when the PZT control voltage has a frequency of 100 Hz, but for different amplitudes, (a)–(c) $V_{PZT}$ is 3.6 V, 8.0 V, and 11.0 V respectively. From Figure 6, it can be found that the number of fringes has a linear relationship with the amplitude of $V_{PZT}$, i.e., about 0.8 fringes per volt, which coincides with our previous analysis.

**Figure 7:**
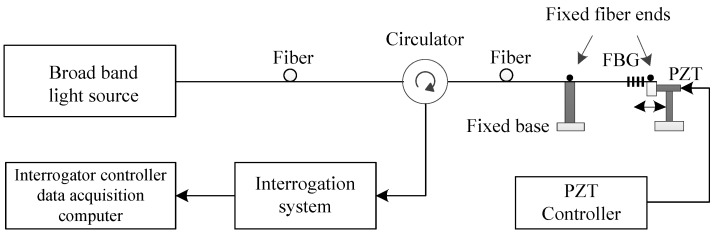
Traditional FBG-based strain measurement system.

**Figure 8:**
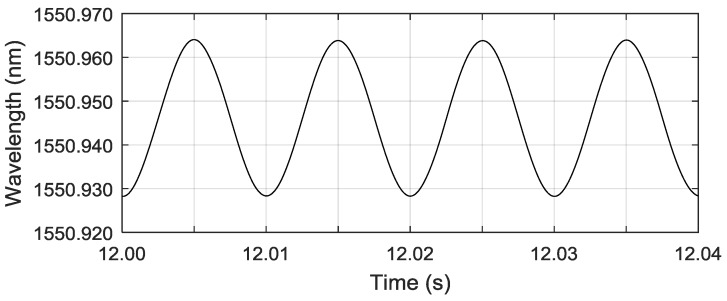
Experiment result by using the I-MON 256 interrogation system.

Then, we made a comparison with a commercial FBG interrogation system (IMON 256, Ibsen Photonics, Farum, Denmark) for dynamic measurement. The system is depicted in Figure 7. A broadband light source is connected to the FBG via a circulator and the reflected signal is directed to the interrogator. Figure 8 shows an example of the measured results by using the commercial interrogation system when the PZT control voltage signal is the same as in Figure 5. As shown in Figure 8, the variation of the Bragg wavelength is in sinusoidal form with a frequency of 100 HZ and a wavelength variation (peak-peak) of 0.035 nm. The strain sensitivity of the typical silica FBG is 1.2 pm/με [29], the strain measured by the interrogation system is 29.2 με, which coincides with the results shown in Figure 5 obtained from the proposed FBG–SMI system.

Regarding the FBG strain caused by the PZT, the value of the strain can be estimated using the PZT data sheet according to the control voltage applied on the FBG, shown in Table 1. We refer these values to test our measurement results obtained from the proposed FBG–SMI system. In addition, we employed the commercial I-MON 256 interrogation system to verify our results. Table 1 presents the comparison for the cases with different strains caused by the PZT. Our results coincide with the ones measured by the commercial system. The above experimental results verify the accuracy of the measurement model in this work. It can be concluded that the FBG–SMI signal can be used for dynamic strain measurements in FBG. With the aid of the algorithm we developed in [31] for recovering the displacement from the SMI signal, a real-time dynamic strain measurement can be obtained. Theoretically, for our SMI–FBG system, the displacement resolution (by fringe counting) is 663 nm, which is equivalent to 4.4 με. The resolution can be further improved by using displacement recovery (e.g., the algorithm reported in [31]) or fringe subdivision. In our experiments, the amplitude of the SMI signal is 60 mV (peak-peak) with a noise of about 3 mV (peak-peak), which means the measurement resolution is 33 nm (0.428*λ*_0_ × 3/60) by using the algorithm in [31], corresponding to a strain of 221 nε. The resolution may be improved by using a denoising algorithm. The above experimental results verify the accuracy of the derived measurement model in this work.

### 3.2. AE Wave Measurement

**Figure 9:**
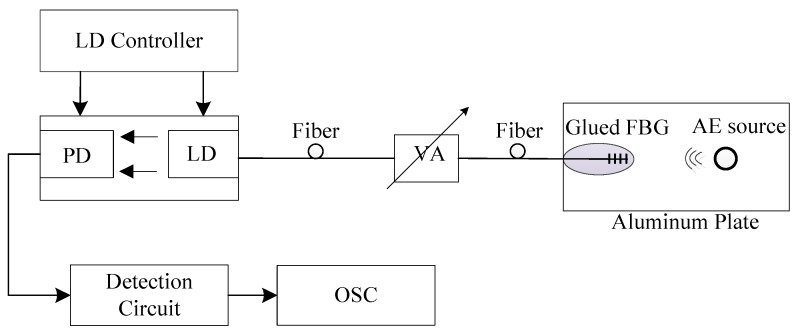
Experimental FBG–SMI system for acoustic emission (AE) measurement.

To demonstrate the feasibility of using the proposed FBG–SMI system for AE measurement, we modified the setup shown in Figure 4 by introducing an AE source and removing the related PZT. The new experiment setup is depicted in Figure 9. The FBG is glued on a 24 × 12 × 0.25 cm aluminum plate using super glue.

**Figure 10:**
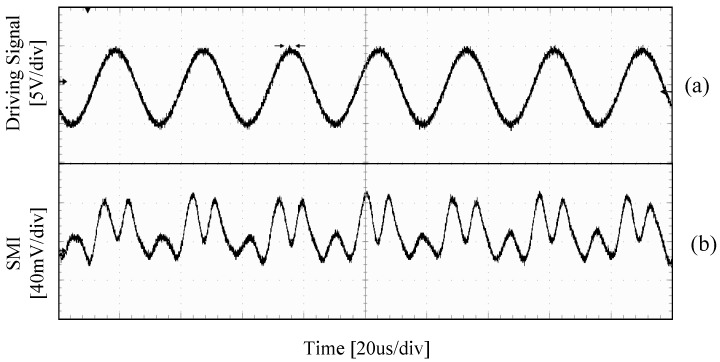
Driving signal on the ultrasonic transducer and its corresponding SMI signal. (**a**) Driving signal for the ultrasonic transducer, (**b**) corresponding SMI signal.

An ultrasonic transducer with a resonant frequency of 40 KHz is adhered on the surface of the aluminum plate to launch ultrasonic waves. The transducer is driven by a customized power driver board. Figure 10a shows the driving signal to the transducer (a sinusoidal signal with frequency of ~38 KHz and amplitude of 10 V). Figure 10b is the corresponding SMI signal captured by our FBG–SMI system. It can be seen from the received SMI signal that about two fringes are generated corresponding to one sinusoidal period of the AE driving signal which indicates that the AE wave induced a displacement of about 665 nm in the FBG.

**Figure 11:**
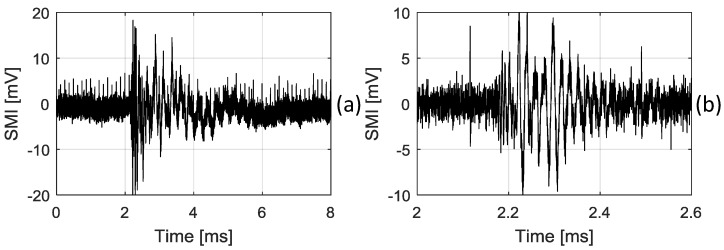
SMI signal corresponding to the AE wave generated by pencil lead breaking, (**a**) a raw detected SMI signal, (**b**) an SMI signal after filtering.

Furthermore, we generated an AE signal by pencil lead breaking, to test out system. In this experiment, the most representative Hsu-Niesen broken lead method was employed, in which the pencil lead had a diameter of 0.5 mm, length of 3.0 mm, and hardness of HB [7]. The position of the pencil lead breaking is 20.0 mm away from the FBG. Figure 11 shows the test result for the AE wave generated by pencil lead breaking. To get a clear AE-induced SMI signal and remove the unwanted low-frequency disturbance, a band-pass filter with bandwidth of 10 KHz–1 MHz [13] is applied on the raw SMI signal shown on Figure 11a and a clear one after filtering is shown in Figure 11b. This experiment demonstrates that the proposed FBG–SMI system is able to capture the pencil-lead-breaking-induced AE wave.

The maximum response speed for an SMI system depends on the photodiode (PD) and its correlated detection circuit. Our current physical system has a bandwidth of 10 MHz, which can meet the maximum frequency requirement for most AE events [1,2,6]. The bandwidth of the system can be further increased by modifying the detection circuit. With the aid of the algorithm we developed in [31] for recovering the displacement from an SMI signal, the AE waveform can be retrieved. The proposed FBG–SMI system is featured with a wide dynamic frequency response and with no need of a broadband light source and expensive interrogation system. It contributes a new low-cost interrogation method for AE measurement by FBG.

## 4. Conclusions

In this work, a new system for detecting AE induced dynamic strain based on SMI and FBG was developed. We first analyzed the SMI theory model for an FBG–SMI system. Then, the preliminary experimental results verified the feasibility of the proposed method. Compared with the existing traditional FBG interrogation system for AE measurement, the FBG–SMI system has a compact structure, resulting in a new low-cost option for FBG-based AE measurement. One point that needs to be noted is that the wavelength of the laser and FBG should match each other to guarantee that the laser can be reflected into the laser cavity. Then the FWHM of the FBG should be wider than that of the laser to guarantee the feedback coefficient C to be close to constant. The readers should be reminded that the influence of the temperature on the FBG has not been discussed in this work. Nonetheless, the proposed sensor in this paper combines the advantages of fiber FGB and SMI, contributing to a novel system in structure health monitoring which can be used to measure AE signals to enable the early detection of failure of structures.

## Figures and Tables

**Table 1:** Comparison of the strain measured by the FBG–SMI system and I-MON 256 system.

PZT (Piezoelectric Transducer) Control Voltage V_PZT_ (V)/freq. (Hz)	Strain Caused by PZT (με)	Strain Measured by FBG–SMI (με)	Strain Measured by I-MON256 (με)
3.6/100	12.7	12.2	12.5
8.0/100	28.3	28.7	29.2
11.0/100	38.9	39.8	38.3
